# Successful Left Atrial Appendage Occlusion with the New Generation Amulet® Device after Late-Occurring Embolization of an Amplatzer® Cardiac Plug in a Patient with Repetitive Strokes

**DOI:** 10.1155/2016/8438923

**Published:** 2016-10-09

**Authors:** Marco R. Schroeter, Wolfgang Schillinger

**Affiliations:** ^1^Department of Cardiology and Pneumology, Heart Center, University of Göttingen, Göttingen, Germany; ^2^Internal Medicine I, Helios Albert-Schweitzer Clinic Northeim, Northeim, Germany

## Abstract

The Amplatzer Cardiac Plug (ACP) is one of the most commonly used devices for percutaneous left atrial appendage (LAA) closure in order to prevent a stroke in patients with atrial fibrillation and contraindication for long-term oral anticoagulation therapy. We have previously described a patient who had experienced an embolization of the ACP device about 12 months after implantation and the device could be percutaneously retrieved. A few years later, he suffered from a posterior stroke and a stroke located in the brainstem as well as a transischemic attack (TIA). In order to protect him from further cardioembolic events a reocclusion of the LAA with the new generation of ACP device, the Amplatzer Amulet, was performed. A stable position of the device within follow-up period could be confirmed and the patient was free of additional strokes/TIA or bleeding events. This case stresses the importance of proper LAA sizing in order to prevent device embolization and notes that LAA size is not static. Moreover, it demonstrates that repeated implantation of an LAA occlusion device was still possible; one should be aware of undersizing the LAA dimensions and that the modifications of new generation LAA occlusion devices may overcome limitations of first-generation devices in order to prevent a cardioembolic stroke.

## 1. Introduction

The Amplatzer Cardiac Plug (ACP; St. Jude Medical) is one of the most commonly used devices for percutaneous left atrial appendage (LAA) closure in order to prevent a stroke in patients with atrial fibrillation and contraindication for long-term oral anticoagulation therapy.

We have previously published a paper describing a 75-year-old patient who had experienced an embolization of the ACP device about 12 months after implantation [1].

Recently, a new generation of ACP device, the Amplatzer Amulet, has been made commercially available. The main concept of the Amulet device is based on the first generation of ACP with a distal lobe and a proximal disk connected by an articulating waist. The lobe has stabilizing hooks to assure retention and the disc seals the outer shape of the LAA orifice [2]. The modifications of the Amulet device include new features to facilitate device deployment and to reduce the embolization risk [3]. The experiences with the new Amulet device are limited so far [3–5].

## 2. Case Report

The above-mentioned patient was discharged at that time after ACP retrieval without an LAA device on an antiplatelet therapy with ASA. Subsequently, he suffered from a posterior stroke and a stroke located in the brainstem as well as a transischemic attack (TIA) in the area of the left cerebri media artery. The patient had been repetitively hospitalized in the stroke-unit/neurological department of our hospital. No relevant extra- or intracerebral stenosis could be detected and a cardioembolic genesis of stroke/TIA caused by atrial fibrillation was presumed. Due to the bleeding risk with the history of intracerebral bleeding, an oral anticoagulation therapy was still contraindicated. In order to protect him from further cardioembolic events we decided to perform reocclusion of the LAA.

**Figure 1:**
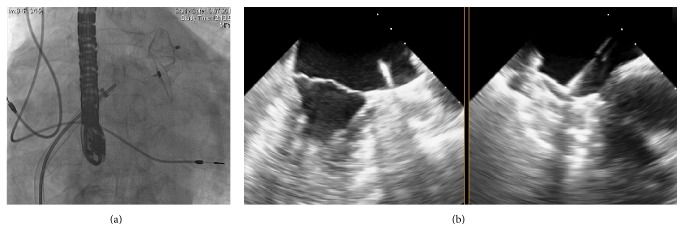
The implantation of the Amulet device was performed under fluoroscopic (a) and echocardiographic (b) guidance, similar to the Amplatzer Cardiac Plug (ACP).

**Figure 2:**
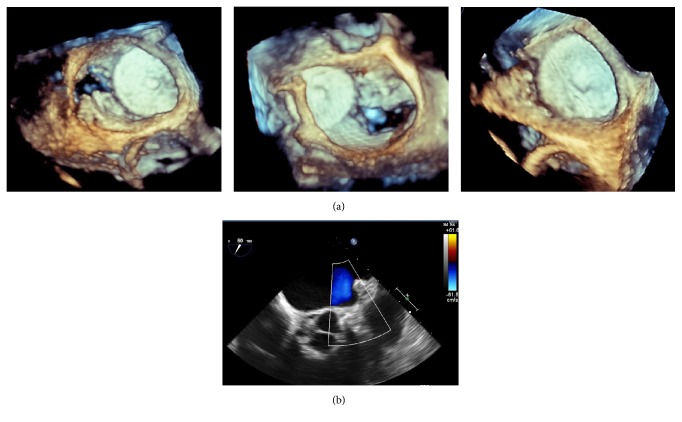
Using 3D transesophageal echocardiography, (a) a stable position of the device within follow-up period could be confirmed (*from left to right*: immediately after implantation, after 3 months and 6 months). Color Doppler TEE revealed complete device occlusion of the LAA (b).

The procedure was performed percutaneously using a transseptal approach under transesophageal echocardiography (TEE) and fluoroscopy guidance (Figure 1). The diameter of a potential “landing zone” in the LAA was unexpectedly found to be greater than during the first intervention: 23 mm (compared to 19 mm measured before) and an Amplatzer Amulet of 28 mm was successfully implanted (Figure 1). After echocardiologic and fluoroscopic control on the next day were inconspicuous, the patient was discharged on an ASA and clopidogrel therapy. Repetitive echocardiographic controls were performed at the date of implantation as well as 3 and 6 months after implantation (3D-TEE; Figure 2(a)). Color Doppler TEE revealed no signs of residual shunt between the LAA and the left atrium (Figure 2(b)). After 3 months the medication was reduced to an ASA monotherapy (100 mg SID) and the patient was free of additional strokes/TIA or bleeding events until now.

## 3. Discussion and Conclusion

The existing case reports and a recently published study with 17 patients described the implantation of the new Amulet device in the normal context of a single implanted LAA device [3–5]. In our case, it was the second device in the same patient after the embolization of the initially implanted ACP device. Subsequently, we had a longer follow-up period with TEE controls compared to previously published reports.

Moreover, this case stresses the importance of proper LAA sizing in order to prevent possible device embolization. It is of note that LAA size is not static. Changes in cardiac preload induce dynamic changes of LAA size. For this reason and with implication for the clinical operation procedure, adequate intravascular volume status must be ensured before final sizing and occluder selection. In addition, dilation of the LAA may occur over time as a result of the remodelling process associated with atrial fibrillation [6]. However, a recently published single case report hypothesizes that LAA device embolization could occur without obvious explanation, despite correct device sizing and sufficient stability tests [7].

In summary, this case demonstrates that repeated implantation of an LAA occlusion device was still possible; one should be aware of undersizing the LAA dimensions (in respect to the volume status of the patient) and modifications of new generation LAA occlusion devices may overcome limitations of first generation, even if further studies with the new devices are necessary.
